# Nodal signaling is required for closure of the anterior neural tube in zebrafish

**DOI:** 10.1186/1471-213X-7-126

**Published:** 2007-11-08

**Authors:** Allisan Aquilina-Beck, Kristine Ilagan, Qin Liu, Jennifer O Liang

**Affiliations:** 1 Department of Biology, Case Western Reserve University, 10900 Euclid Avenue, Cleveland, OH USA; 2 Department of Biology, University of Akron, 302 Buchtel Common, Akron, OH USA; 3 Department of Genetics, Case Western Reserve University, 10900 Euclid Avenue, Cleveland, OH USA

## Abstract

**Background:**

Nodals are secreted signaling proteins with many roles in vertebrate development. Here, we identify a new role for Nodal signaling in regulating closure of the rostral neural tube of zebrafish.

**Results:**

We find that the neural tube in the presumptive forebrain fails to close in zebrafish Nodal signaling mutants. For instance, the cells that will give rise to the pineal organ fail to move from the lateral edges of the neural plate to the midline of the diencephalon. The open neural tube in Nodal signaling mutants may be due in part to reduced function of N-cadherin, a cell adhesion molecule expressed in the neural tube and required for neural tube closure. N-cadherin expression and localization to the membrane are reduced in fish that lack Nodal signaling. Further, N-cadherin mutants and morphants have a pineal phenotype similar to that of mutants with deficiencies in the Nodal pathway. Overexpression of an activated form of the TGFβ Type I receptor Taram-A (Taram-A*) cell autonomously rescues mesendoderm formation in fish with a severe decrease in Nodal signaling. We find that overexpression of Taram-A* also corrects their open neural tube defect. This suggests that, as in mammals, the mesoderm and endoderm have an important role in regulating closure of the anterior neural tube of zebrafish.

**Conclusion:**

This work helps establish a role for Nodal signals in neurulation, and suggests that defects in Nodal signaling could underlie human neural tube defects such as exencephaly, a fatal condition characterized by an open neural tube in the anterior brain.

## Background

Nodals are secreted signaling proteins in the TGFβ superfamily that have many established roles in vertebrate development. The absence of Nodal signaling in mice and zebrafish results in loss of the gastrula organizer, an almost complete failure in the development of mesodermal and endodermal tissue, and severe defects in the cellular movements of gastrulation [1,2]. Over expression and partial loss-of-function studies indicate that Nodal signaling not only induces mesoderm, but also acts in a concentration dependent fashion to subdivide the mesoderm into different tissue types [3]. Further, Nodal signaling in the left lateral plate mesoderm has a conserved role in regulating left-right asymmetry of visceral organs such as the heart and lungs [4].

Nodal signals also have extensive roles during neural development. Nodal proteins cooperate with several other signaling proteins, including Bone Morphogenetic Protein (BMP), Fibroblast Growth Factor (FGF), and Wingless Integrated (Wnt) to regulate anterior-posterior patterning of the neurectoderm [5-11]. Nodal signals are also important for patterning the ventral neural tube: lack of the Nodal signal Cyclops (Cyc) in zebrafish results in a complete absence of ventral brain and a severe reduction of the floor plate cells in the ventral spinal cord [12-15]. Because Nodal signaling mutants lack the Sonic hedgehog (Shh) expressing cells of the ventral brain and prechordal plate, they often have holoprosencephaly, or a failure in the forebrain to bifurcate into two hemispheres [16]. In zebrafish, Nodal signals also regulate laterality in the dorsal forebrain. Several genes in the Nodal signaling pathway are expressed on the left side of the developing pineal organ, where they influence left-right asymmetry of the habenula nuclei and the pineal complex, which consists of a medial pineal organ and a left-sided parapineal [17-21].

Zebrafish have three Nodal signals, Cyc, Squint (Sqt), and Southpaw (Spaw), which all function through a receptor complex containing the membrane associated protein One-eyed pinhead (Oep). Here, we demonstrate that Sqt and Oep are important for closure of the anterior neural tube. During development, pineal precursors originate in two domains at the lateral edges of the neural plate [22-27]. As development proceeds, these precursors converge towards the midline of the dorsal diencephalon, where they ultimately fuse to form a single pineal organ. Thus, the position of the pineal precursors serves as a sensitive measure of neural tube closure. We find that the pineal precursors often fail to converge to the midline in *sqt *mutants, resulting in a pineal anlage that is elongated laterally or divided into two domains. In maternal zygotic *oep *(MZ*oep*) mutants, which lack both maternally derived and zygotically expressed *oep *mRNA, the pineal precursors remain in two widely separated domains. The cell adhesion molecule N-cadherin (N-cad) is required for closure of the neural tube in zebrafish [28,29]. Our data suggests that Nodal signaling may influence neural tube closure by regulating N-cad. N-cad protein localization to the cell membrane was reduced in MZ*oep *mutants, and the structure of the neural tube was severely disrupted. We found that cell autonomous rescue of mesendoderm (a tissue that gives rise to both mesodermal and endodermal cell types) in MZ*oep *mutants corrected their neural tube defect (NTD). This suggests that the role of Nodal signaling is to induce mesoderm formation, and mesoderm in turn regulates neural tube closure. This role of mesoderm may be conserved, as mesoderm also has a key role in rostral neural tube closure in mice [30]. NTDs such as spina bifida and exencephaly occur in approximately one in one thousand human births, but the genetic causes of these disorders are still poorly understood [31]. This work raises the possibility that deficiencies in the Nodal signaling pathway could underlie defects in closure of the rostral neural tube in humans.

## Results

### Nodal signaling is required for dorsal convergence of pineal precursors

**Figure 1:**
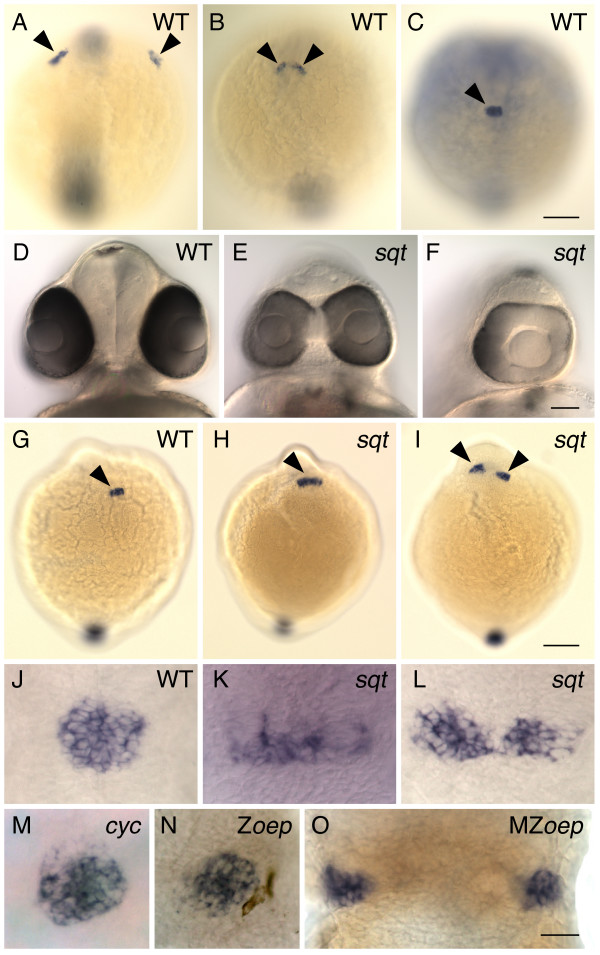
**Pineal precursors fail to reach the midline of the forebrain in *squint *mutants**. (A-C) WT embryos were processed for whole mount in situ hybridization with an antisense probe for the gene *flh*, which is expressed in the pineal precursors (arrowheads). Images are dorsal views of the entire embryo, anterior to the top. (A) At the 2–3 somite stage, pineal precursors are located in two widely spaced lateral domains. (B) By the 5–6 somite stage, these domains have moved towards the dorsal midline of the forebrain. (C) By the 7–8 somite stage, a single, round-shaped pineal anlage has formed. (D-F) At 2 dpf, *sqt *mutant embryos from the same clutch have a wide range of eye phenotypes. Frontal views of live embryos with dorsal to the top. (D) The eyes of WT embryos and some *sqt *mutants (not shown) are completely separated from one another. (E) Other *sqt *mutants have partially fused eyes that form two lenses or (F) a single eye with one lens. (G-L) Embryos at the (G-I) 7–8 somite stage and (J-L) 24 hpf were processed for whole mount in situ hybridization with a probe for the pineal gene (G-I) *flh *or (J-O) *otx5*, dorsal views, anterior to the top. (G,J) In WT siblings, the pineal precursors (arrowhead) have converged to form a round pineal anlage. In *sqt *mutants the pineal precursors (arrowheads) form a domain that is (H,K) elongated or (I,L) divided in two. The pineal anlagen of the (M) *cyc *mutant and the (N) Z*oep *mutant have a round shape that is similar to that of WT fish, while the pineal precursors of the (O) MZ*oep *mutant are divided in two domains. All images are dorsal views with anterior to the top. Scale bars: 100 μm (A-C, G-I), 70 μm (D-F), 30 μm (J-O).

In WT zebrafish, *floating head *(*flh*) expressing pineal precursors are initially located in two widely separated domains on either side of the neural plate (Figure 1A)[26]. As development proceeds, these domains converge towards the dorsal midline of the brain, and fuse to form a single, round-shaped pineal anlage at the midline of the dorsal diencephalon (Figure 1B, C)[26]. Through a screen of existing mutants for defects in pineal morphology, we found that convergence of the pineal precursors to the midline of the brain is disrupted in zebrafish that lack the Nodal signal Sqt. At the 7–8 somite stage and at 1 day post fertilization (dpf), the domain encompassing the pineal precursors in homozygous *sqt *mutants could be indistinguishable from WT, elongated laterally, or divided into two domains (Table 1, Figure 1G–L). This variability has also been found in other aspects of the *sqt *phenotype. Some homozygous *sqt *mutants are indistinguishable from WT siblings and live to adulthood, while the most severely affected are cyclopic and have significant loss of ventral brain and mesodermal tissues (Figure 1D–F)[32].

**Table 1:** Embryos that lack Nodal signaling or N-cad expression have defects in the convergence of pineal precursors

	Pineal morphology		
	
Embryo	Normal	Expanded	Divided
*sqt*^*cz*35^/+ X *sqt*^*cz*35^/+			
cyclopic eye	90	45	61
normal eyes*	814	3	1
			
*cyc*^*m*294^	140	0	0
WT siblings	343	0	0
			
Z*oep*^*m*134^	83	0	0
WT siblings	157	0	0
			
MZ*oep*^*m*134^	0	5	117
			
*pac*^*p*79*m*^	58	18	10
WT siblings	191	0	0
			
*glo*^*m*117^	13	17	28
WT siblings	85	0	0

Embryos were fixed at 1–3 dpf (for *sqt*), 1 dpf (for *cyc*, Z*oep*, MZ*oep*, and *pac*), or 2–4 dpf (for *glo*), and then processed for whole mount in situ hybridization with a pineal specific probe (*otx5*, *flh*, *gnat1*, *gnat2*) or antibody staining with the pineal photoreceptor specific antibody zpr1.

*Embryos with two normal eyes could be homozygous WT, heterozygous for *sqt*^*cz*35^, or homozygous for *sqt*^*cz*35^.

**Figure 2:**
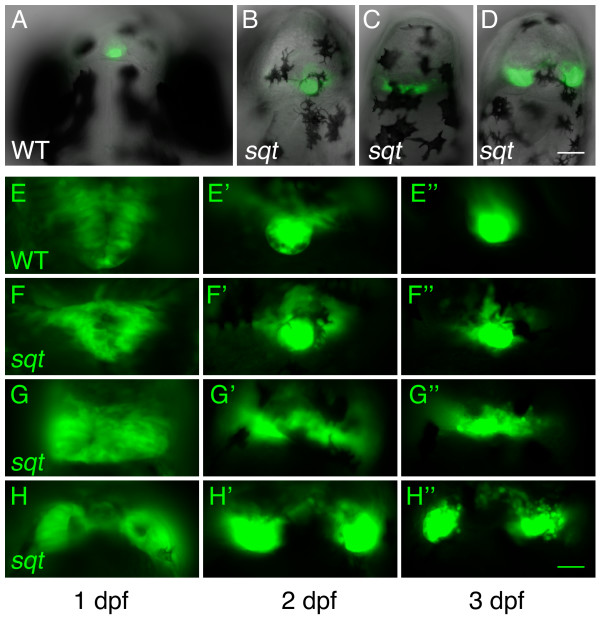
**Elongated and divided phenotypes in *squint *mutants persist through the first three days of development**. The morphology of the pineal was followed over time in individual *sqt *mutants and WT siblings carrying the *flh*:eGFP transgene, which expresses GFP throughout the pineal anlage during embryogenesis [21]. All *sqt *mutants used in this experiment had cyclopic eyes. (A-D) Composite bright field and fluorescent images at 2 dpf showing fluorescence in the pineal anlage of embryos having (A-B) normal, round-shaped pineal anlagen, and (C) elongated and (D) divided pineal anlagen. (E-H") Each row shows the pineal of an individual embryo over three days of development as assayed by fluorescence microscopy on a compound microscope. Note the similarity in the morphology of the pineal in (E-E") the WT embryo and (F-F") one of the *sqt *mutants. In contrast, the *sqt *mutants in G-G" and H-H" maintain their abnormal elongated and divided morphologies throughout the experiment. All images are dorsal views with anterior to top. Scale bars: 60 μm (A-D), 30 μm (E-H").

To characterize the phenotype of *sqt *mutants more fully, morphology of the developing pineal was followed over time in individual WT and *sqt *embryos carrying the *flh*:eGFP transgene, which drives eGFP expression in the pineal [21]. Homozygous *sqt *mutants were unambiguously identified by their cyclopic eye phenotype. Siblings with two normal eyes could have had one of three genotypes; *+*/*+*, *sqt*/*+*, or *sqt*/*sqt*. Fish with normal, elongated, and divided pineal phenotypes were identified by fluorescence microscopy at 1 dpf and then followed for the next two days (Figure 2). In *sqt *mutants with a round shaped pineal at 1 dpf (Figure 2B, and 2F–F"), the pineal anlage remained indistinguishable from their WT siblings through the following two days (Figure 2E–F"). In *sqt *mutants with an elongated or divided pineal at 1 dpf (Figure 2C, D, and 2G–H"), the abnormal pineal morphology persisted or became even more severe over time (Figure 2G–H"). If the elongated/divided pineal phenotypes were merely due to a delay in development, then they should have been rectified over the three days of the experiment. The persistence of these phenotypes suggests that they are instead due to a defect in the mechanism that drives convergence of the pineal precursors to the midline of the diencephalon.

To more fully define the role of Nodal signaling in the movement of pineal precursors, we examined pineal morphology in other Nodal signaling mutants. The phenotype of mutants that lack Cyc, another zebrafish Nodal signal, includes a partial loss of mesendodermal tissue, lack of ventral brain and floor plate, and randomized laterality of the pineal complex and habenula nuclei [14,17,19,33]. In contrast to *sqt*, the pineal precursors in 1 dpf *cyc *mutants always formed a single pineal anlage with a round morphology, suggesting that the pineal precursors had successfully moved to the midline of the brain (Table 1, Figure 1M).

Cyc and Sqt signal through a common receptor complex that contains the protein Oep, a member of the EGF-CFC family [34]. Maternally derived *oep *mRNA is loaded into the oocyte and then later transcribed from the zygotic genome [35]. Mutants that lack only zygotic *oep *(Z*oep*) have an overall phenotype that is very similar to *cyc *mutants. For instance, both Z*oep *and *cyc *mutants lack ventral brain but have a fairly normal notochord, while the most severe *sqt *mutants lack both notochord and ventral brain [10,36-39]. Consistent with the other similarities between *cyc *and Z*oep*, we found that Z*oep *mutants also had a single, round pineal anlage (Table 1, Figure 1N).

In MZ*oep *embryos, which lack both maternal and zygotic *oep *mRNA, the ventral neural tube, endoderm, and most mesoderm fail to develop [34,40]. Despite these severe defects, anterior-posterior patterning of the brain is fairly normal and the pineal precursors are specified [17,19,34]. However, we found that pineal development was severely affected in MZ*oep *fish: the pineal precursors were found in two widely spaced domains, or in rare cases, in a single, elongated domain (Table 1, Figure 1O).

Together, this suggests that Sqt signaling through a receptor complex containing Oep is important for the convergent movements of the pineal precursors. The fact that the pineal phenotype of MZ*oep *mutants is much more severe than most *sqt *mutants suggests that loss of zygotically expressed *sqt *is in part compensated for by one of the other zebrafish Nodal signals or by Sqt translated from maternal mRNA. This is not unexpected, as there is partial redundancy between the three zebrafish Nodal signals in other aspects of their functions, such as patterning the endoderm and mesoderm [41].

### The Nodal signaling mutants *sqt *and MZ*oep *have an open neural tube

During development, the flat neural plate epithelium folds or rolls to form the neural tube, driving convergence of the pineal precursors to the midline of the brain. This suggests that the elongated/divided pineal phenotypes are due to a failure in neurulation. To test this, we took advantage of the variable pineal phenotype in *sqt *mutants. If this hypothesis is correct, then the neural tube should be open only in *sqt *mutants with an elongated or divided pineal morphology.

**Figure 3:**
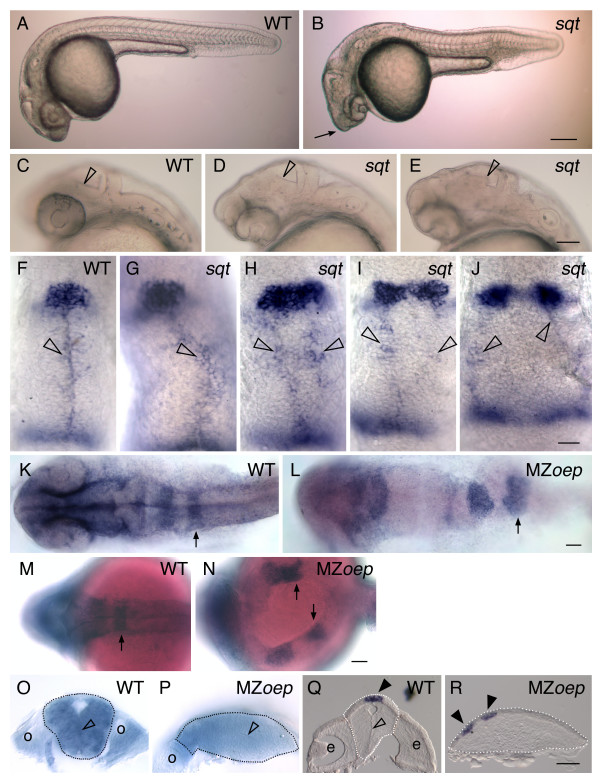
**Correlation between an expanded or divided pineal and an open neural tube**. (A-E) Lateral views of live embryos at 1 dpf, anterior to the left. (A) While the head of the WT embryo is smooth and rounded, (B) the head of the *sqt *embryo is pointed (arrow). (C-E) Higher magnification of the anterior embryo reveals variability in the brain morphology of *sqt *mutants. In (C) WT embryos and (D) some *sqt *mutants, the border between the tectum and tegmentum (open arrowheads) appears as a smooth, straight line. (E) However, in some *sqt *mutants the border appears to be abnormally shaped or indistinct (open arrowhead), suggesting that the morphology of tectum or tegmentum is perturbed. (F-J) Embryos were fixed at 1 dpf, and processed for in situ hybridization with antisense probes for the pineal gene *otx5 *and the dorsal neural tube gene *wnt1*. In (F) WT and (G) *sqt *embryos with a single, round pineal anlage, the *wnt1 *expressing cells (open arrowheads) form a single domain along the dorsal neural tube. In contrast, *sqt *embryos with an (H) elongated or (I-J) divided pineal anlage have two parallel lines of *wnt1 *expressing cells. (K-P) Embryos were fixed at 1 dpf, processed for in situ hybridization with an antisense probe for *epha4a*, and then either (K-N) imaged in dorsal view, anterior to the left or (O, P) cut through *epha4a*-expressing rhombomere 5 to bisect the embryo into anterior and posterior halves. The locations of the otic vesicles (o), rhombomere 5 (arrows), and midline (open arrowhead) are indicated. A potential region of midline is marked by the open arrowhead in P. (Q, R) 14 μM frozen cross sections through the diencephalon of 1 dpf (Q) WT or (R) MZ*oep *embryos stained for *otx5 *expression. The midline of the brain (open arrowhead), and pineal precursors (closed arrowheads) are indicated. Dotted lines outline the neural tubes in panels O-R. Scale bars: 200 μm (A,B), 100 μm (C-E), 30 μm (F-J), 50 μm (K-R).

Since *sqt *mutants have not been previously shown to have a neural tube closure defect, we examined the neural tube by morphology in live embryos and through the expression of molecular markers. *sqt *mutants often had a "pinhead" appearance characteristic of other Nodal signaling mutants (such as *oep*) (Figure 3A,B), and a tectum and tectal ventricle that appeared disordered (Figure 3C–E).

To determine whether the anterior neural tube was open in *sqt *mutants with pineal defects, embryos were analyzed for *wingless integrated 1 *(*wnt1*), which is expressed in the cells at the converging edges of the neural plate that will ultimately become the roof plate [29,42,43], and *orthodenticle homeobox *5 (*otx5)*, which is expressed in the developing pineal [20]. WT embryos at 24 hours post fertilization (hpf) had a single domain of *wnt1 *along the dorsal neural tube and a round shaped pineal organ (Figure 3F). *sqt *mutants with a round pineal also had a single stripe of *wnt1 *staining, although it undulated (Figure 3G). In contrast, *sqt *mutants with either an elongated or divided pineal domain always had two parallel lines of *wnt1 *expression, demonstrating that the presumptive roof plate cells had not converged to the midline (Table 2, Figure 3H–J). MZ*oep *mutants also had an open neural tube in the anterior region (Table 2 and data not shown). Thus, there is a perfect correlation between an open neural tube and the failure of pineal precursors to converge at the midline of the brain (Table 2). In contrast, there was no correlation between the severity of the pineal and eye phenotypes: some *sqt *mutants with cyclopic eyes had an apparently normal, round pineal anlage (Table 1, Figure 2).

**Table 2:** Correlation between pineal and neural tube phenotypes in Nodal signaling mutants

	Neural tube morphology	
	
Embryo	Closed	Open
*sqt*^*cz*35^/+ X *sqt*^*cz*35^/+		
normal pineal	353*	0
elongated/divided pineal	0	11
		
MZ*oep*^*m*134^	0	55

Embryos were fixed at 1 dpf and then assayed for expression of *otx5 *and *wnt1*. Pineal and neural tube phenotypes were scored from a dorsal view.

*Embryos with a normal pineal morphology are a mixture of WT and *sqt *mutant embryos.

The neural tube defect in MZ*oep *mutants was further characterized through examination of *ephrina4a *(*epha4a*) expression in the hindbrain. In WT embryos, *epha4a *was expressed in three stripes that correspond to rhombomeres 1, 3 and 5 (Figure 3K, M)[44]. In some MZ*oep *embryos *epha4a *was expressed in a similar pattern to WT embryos, except that the rhombomeres were somewhat misshapen and slightly wider along the anterior posterior axis (Figure 3L). In approximately half of the MZ*oep *embryos, the hindbrain was severely disrupted, including partial fusion of rhombomeres 3 and 5 and apparent separation of the left and right sides of the neural tube (Figure 3N).

Transverse sections through rhombomere 5 and the diencephalon further demonstrated the extent of the neural tube phenotype in MZ*oep*. The neural tube at both axial levels of WT embryos was symmetric with a small lumen at the dorsal side (Figure 3O, Q). The MZ*oep *neural tube was shortened along the dorsal-ventral axis and the midline was not apparent (Figure 3P, R). As we have previously reported, the brain of MZ*oep *is often twisted, and the pineal precursors were often located on one side of the developing brain (Figure 3R) [19].

### Pineal precursors fail to converge normally in embryos that lack N-cad

**Figure 4:**
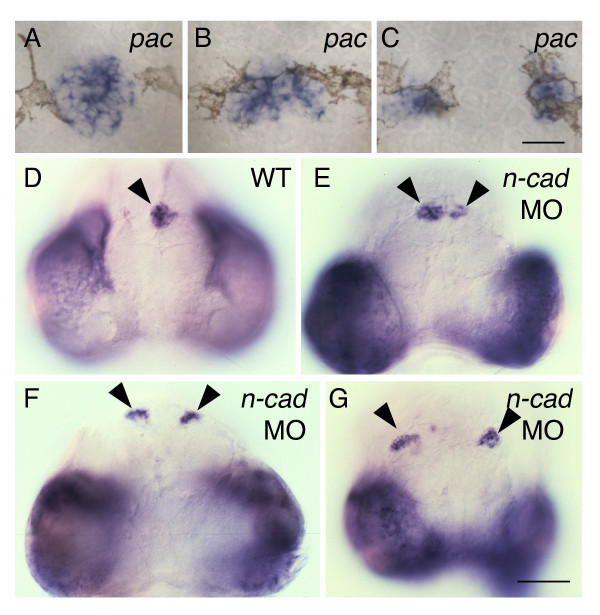
**The expanded and divided pineal phenotype is present in *n-cad *mutants and morphants**. (A-C) Homozygous *pac *mutants were fixed at 30 hpf, and then processed for in situ hybridization with an antisense probe for *otx5*. Dorsal views with anterior to the top. *pac *mutants could have a (A) normal, round-shaped pineal morphology, (B) an elongated pineal anlage or (C) a divided pineal. (D-G) Embryos were injected at the one to four cell stage, fixed at 74 hpf, and then assayed for expression of *otx5*. Frontal views with dorsal to the top. The presumptive pineal organ (arrowheads) forms (D) a single domain in control embryos and a (E-G) divided pineal in embryos depleted in N-cad through morpholino (MO) injections. Scale bars: 30 μm (A-C), 100 μm (D-G).

The cell surface protein N-cad is required for adhesion between cells of the zebrafish neuroepithelium. In zebrafish *parachute *(*pac)/n-cad *mutants, the convergent extension movements that shape the neural plate are altered and the dorsal neural tube fails to close [28,29]. Expression of *otx5 *in *pac *mutants revealed a strikingly similar range of pineal phenotypes to those found in *sqt *mutants. The presumptive pineal could be round, elongated, or divided (Figure 4A–C). The abnormal pineal phenotypes were found at a higher rate in *glass onion *(*glo*) embryos, which are more severely affected *n-cad *mutants (Table 1). Further, antisense morpholino (MO) mediated depletion of N-cad resulted in a divided pineal phenotype (Figure 4D–G). The presence of the divided pineal in *pac/n-cad/glo *mutants and *n-cad *morphants suggests there could be a connection between Nodal and N-cad.

### N-cad expression is altered in the MZ*oep *neural tube

**Figure 5:**
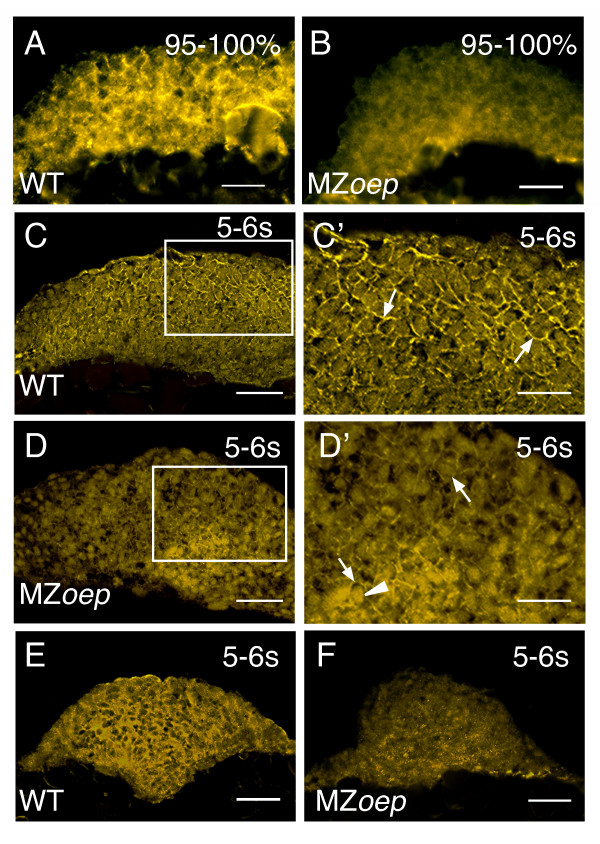
**N-cad expression in the anterior neural keel is altered in MZ*oep *mutants**. (A-F) N-cad immunostaining of tissue sections from WT and MZ*oep *embryos. (A, B) Sagittal sections though the anterior neural keel with anterior to the left and dorsal to the top. (C-D') Parasagittal sections with anterior to the left and dorsal to the top. Panels C' and D' are higher magnifications of the boxed regions in panels C and D, respectively. Arrows point to N-cad labeling in the cell membrane, while the arrowhead indicates labeling in the cytoplasm. (E, F) Transverse sections through the anterior neural tube, dorsal to the top. n ≥ 30 embryos for each sample. Embryos are at 95–100% epiboly (95–100%) or the 5–6 somite stage (5–6 s). Scale bars: 40 μm (C, D, E, F), 20 μm (A, B, C', D').

To explore the relationship between Nodal signaling and cell adhesion, N-cad expression was compared between MZ*oep *and control embryos. Age-matched controls were produced by mating rescued *oep/oep *females with *oep*/+ males, resulting in clutches of eggs that were 50% *oep*/+ and 50% MZ*oep*, or by comparing clutches of WT and MZ*oep *embryos that were fertilized at the same time. No change in the levels of *n-cad *mRNA could be detected between MZ*oep *and WT embryos processed in parallel for whole mount in situ hybridization (data not shown). However, N-cad immunostaining on tissue sections showed that protein expression patterns were altered (Figure 5).

Expression of N-cad protein can be first detected at low levels around the onset of gastrulation. Levels increase and are quite high by the end of epiboly [45]. Consistent with this, we find that N-cad levels were low at 60% epiboly, and no differences could be found between WT and MZ*oep *(data not shown). In contrast, at 95–100% epiboly, just before neurulation begins, MZ*oep *embryos had significantly reduced levels of N-cad protein (5A, B). In particular the characteristic N-cad staining at the cell membrane was clearly apparent in the WT embryo, but nearly absent in the mutant tissue.

The decreased levels of N-cad localization at the membrane were also present at the six somite stage, when the anterior neural tube is just closing to form a neural rod. In WT embryos, N-cad expression was strong and almost evenly distributed in the head region (Figure 5C, E). In *MZoep *embryos, N-cad staining was reduced in most regions except in clusters of cells in deeper (ventral) regions of the tissue (Figure 5D, F). Closer examination of the tissue sections revealed that N-cad expression in the WT tissue was confined mainly to the membranes of cells (Figure 5C'). In contrast, labeling in the plasma membrane of MZ*oep *embryos was much reduced in most cells, similar to the phenotype at 100% epiboly (Figure 5D', F). N-cad expression in cells located in the clusters with strong N-cad expression was either inside the cell and on the cell membrane, or mainly inside the cell (Figure 5D'). This suggests that Nodal signaling during early development could directly or indirectly regulate the expression levels and/or intracellular localization of N-cad.

### The cellular organization of the neural tube is perturbed in MZ*oep *mutants

If the neural tube defect in MZ*oep *mutants is in part due to the abnormal expression of N-cad, then MZ*oep *embryos should display similar cellular phenotypes to those found in *n-cad *mutants. These include decreased cell adhesion and failure of the dorsal/lateral cells to lengthen and intercalate to form a single cell layer [28]. To determine whether MZ*oep *mutants displayed similar cellular phenotypes, we injected one cell stage embryos with mRNA encoding membrane bound GFP (mGFP) and assayed neural tube morphology at several points during neurulation.

**Figure 6:**
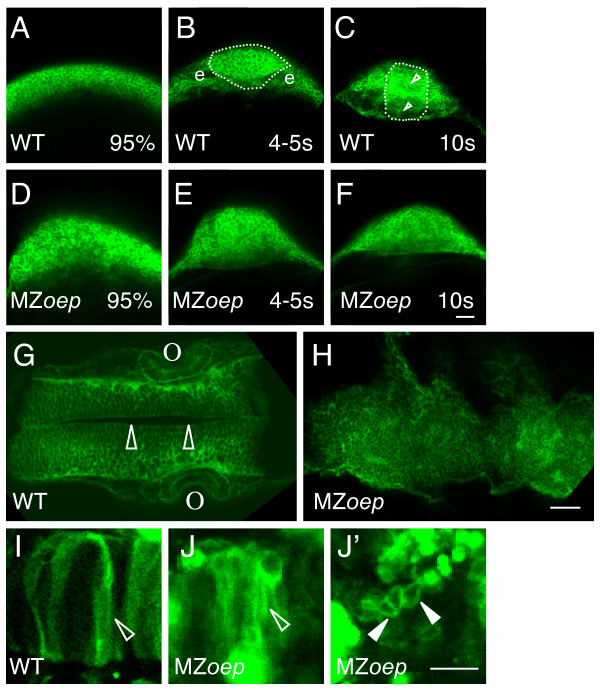
**The ordered structure of the neural tube is disrupted in MZ*oep *embryos**. (A-H) Embryos were injected at the one cell stage with mGFP mRNA and imaged (A-F) live at 95% epiboly (95%), the 4–5 somite stage (4–5 s), the 10 somite stage (10 s), or (G, H) fixed at ~24 hpf. (A-F) Cross sections through the anterior developing neural plate, with presumptive eyes (e) indicated in panel B, the outer boundary of the neural tube indicated by a dotted line in panels B and C, and the midline indicated by the open arrowheads in C. (G, H) Horizontal sections through the midbrain and hindbrain of ~24 hpf embryos, with the midline of the brain (open arrowheads) and the otic vesicles (o) indicated in panel G. (I-J') Embryos were injected at the one cell stage with DNA encoding mGFP, raised to ~24 hpf, and imaged live in high magnification horizontal sections. Open arrowheads indicate elongated cells and the closed arrowheads indicate round cells. All images are confocal optical sections. Scale bars: 40 μm (A-H), 80 μm (I-J').

Consistent with previous studies, the neuroectoderm of WT embryos at 95% epiboly is in the form of a flat neural plate (Figure 6A). At the 4–5 somite stage, folding of the neural tube had started, giving the neuroepithelium the half circle appearance typical of the neural keel stage (Figure 6B). By the 10 somite stage, a round neural rod with a clear midline and a defined outer edge had formed (Figure 6C).

In MZ*oep *embryos, convergence of cells towards the dorsal side occurs normally, but involution of these cells to form the mesendoderm is severely disrupted [34]. As a result, the precursors to the neural tube accumulate at the dorsal margin. Cross sections through this region revealed cells that appeared similar to those in WT embryos, although the tissue was significantly thicker (Figure 6D). Although the presumptive neural tube of MZ*oep *mutants changed morphology and became "rounder" as the embryos developed to the five and ten somite stages, the boundaries of the neural tube remained indistinct and no midline could be identified (Figure 6E, F).

At 24 hpf, dorsal views of WT embryos revealed their highly ordered neuroepithelium and a distinct midline (Figure 6G). In contrast, the developing neural tube of MZ*oep *embryos was crooked (Figure 6H), consistent with the undulating pattern of *wnt1 *staining in *sqt *mutants (Figure 3G). Further, there continued to be no apparent order to the arrangement of cells (Figure 6H). As was previously found in our cross sections (Figure 3P, R), no midline could be discerned (Figure 6H).

The morphology of cells within the neural tube was characterized by injection of mGFP DNA, which labels only a subset of cells and makes it easier to trace cell shape [28]. The WT neural tube contained many elongated cells contacting the midline of the developing brain (Figure 6I)[28,29,46,47]. Elongated cells were also present in the anterior neural tube of MZ*oep *mutants (Figure 6J). However, there were also regions where the cells had a round morphology (Figure 6J'). The phenotypes present in the MZ*oep *neural tube are consistent with the presence of a cell adhesion defect that inhibits formation of an ordered epithelium.

### Injection of N-cad mRNA corrects the NTD in a small percentage of MZ*oep *fish

If lack of N-cad expression and decreased cell adhesion is an important cause of the NTD in Nodal signaling mutants, then overexpression of N-cad protein might be able to correct the NTD. To test this hypothesis, we overexpressed N-cad by injecting mRNA encoding *Xenopus *N-cad into embryos at the one cell stage. In WT embryos, these injections caused an overexpression phenotype similar to that described by Bitzur and colleagues, suggesting that the *Xenopus *protein was active in zebrafish (Additional file 1) [45].

**Figure 7:**
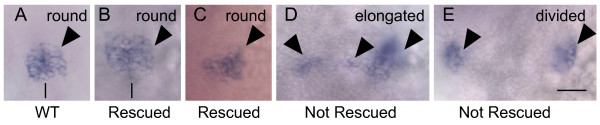
**Injection of n-cad mRNA into MZ*oep *embryos**. Embryos were injected at the one cell stage with n-cad mRNA, raised to approximately 24 hpf, and fixed and processed for whole mount in situ hybridization with a probe for *otx5*. (A) WT and (B, C) some N-cad overexpressing MZ*oep *embryos have a round shaped pineal morphology indicative of a closed pineal organ. (D, E) Other N-cad overexpressing MZ*oep *fish have an elongated or divided pineal organ, demonstrating that their NTD has not been corrected. All images are dorsal views with anterior to the top. The background in Panel C has a pink cast because the embryo was too fragile to remove from the yolk. The midline of the brain is apparent in panels A and B, and is indicated by a black line. Scale bar: 40 μm (A-E).

The large majority of N-cad overexpressing MZ*oep *mutants maintained an abnormal pineal phenotype, indicating that the neural tube had not closed (Figure 7D, E, Table 3). However, in rare cases (n = 3/106) the MZ*oep *pineal had a round shaped pineal morphology (Figure 7B, C). In the most striking case, the midline of the brain was also apparent (Figure 7B). This suggests that loss of N-cad is at least one of the important causes of the NTD in MZ*oep *fish.

**Table 3:** Injection of N-cad mRNA does not correct the NTD in MZ*oep *mutants

		Pineal morphology		
		
Embryo	Dose of N-cad mRNA (pg)	Round	Expanded	Divided
WT	0	75	0	0
WT	1.9–75	37	0	0
MZ*oep*^*m*134^	0	0	1	29
MZ*oep*^*m*134^	0.6–7.5	2	11	58
MZ*oep*^*m*134^	25–75	1	1	33

Embryos were fixed at approximately 1 dpf and processed for whole mount in situ hybridization with an antisense probe to *otx5*.

### Overexpression of Taram-A* rescues mesendoderm formation and neural tube closure in MZ*oep *mutants

**Figure 8:**
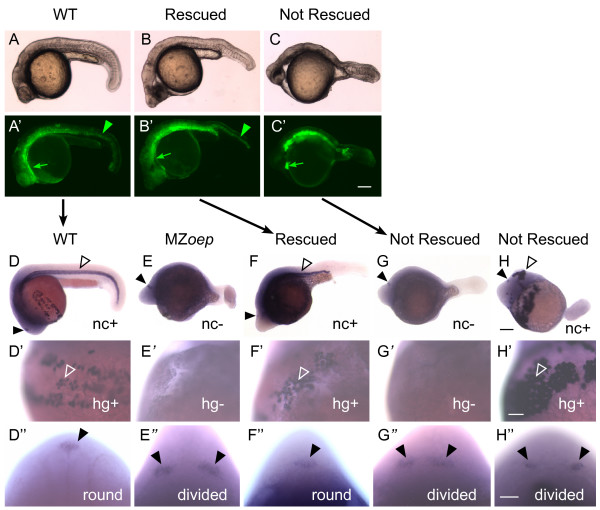
Taram-A* expression results in recovery of mesendoderm and neural tube closure in MZ*oep *mutants. Embryos were co-injected with Taram-A* and mGFP mRNA into one blastomere at the 16 cell stage, raised until 24 hpf, and imaged (A-C') live by (A-C) bright field and (A'-C') fluorescent microscopy, and then (D-H") fixed and processed for whole mount in situ hybridization with *otx5 *to mark the pineal, *ctsl1b *to mark the hatching glands (hg), and *col2a1a *to mark the notochord (nc). Panels with the same letter are different views of the same embryo. In addition, the embryos shown in the A, B, and C rows are the same embryos as in the D, F, and G rows, as indicated by the black arrows. (A'-C') Green arrows indicate fluorescence in the anterior mesendoderm, green arrowheads fluorescence in the notochord. (D-H) Closed arrowheads indicate the pineal organ, and open arrowheads the notochord. Whether an embryo is positive (+) or negative (-) for notochord staining is indicated. (D'-H') Open arrowheads point to hatching gland cells, and whether the embryo is positive or negative for these cells is indicated as in D-H'. (D"-H") Closed arrowheads point to the regions of pineal precursors, and the morphology of the pineal anlage is noted. (A-H') Sagittal views with anterior to the left. (D"-H") Dorsal views of the back of the head. Scale bars: 100 μm (A-H), 50 μm (D'-H").

In mice, mesoderm has a key role in driving the closure of the anterior neural tube [30]. Thus, one possibility is that the neural tube defects in *sqt *and MZ*oep *mutants is due to their deficiencies in mesendoderm formation. To test this we overexpressed Taram-A*, an activated form of a zebrafish Type I TGFβ receptor, by injecting Taram-A mRNA into one blastomere of 16 cell stage MZ*oep *embryos. This method induces a subset of the embryo's cells to adopt a mesendodermal fate by activating the Nodal signaling pathway within these cells (Figure 8A–F) [12,48-51]. Embryos were then raised to approximately 24 hpf and assayed for expression of *cathepsinL 1b *(*ctsl1b*) in the mesendoderm-derived hatching gland cells, *collagen type II alpha 1a *(*col2a1a*) in the mesoderm-derived notochord, and *otx5 *in the pineal (Figure 8D–H", Table 4).

**Table 4:** Expression of Tarama* rescues mesoderm formation and neural tube closure

		Pineal morphology		
		
Embryo	MRNA	Round	Expanded	Divided
MZ*oep*^*m*134^	None			
hg-; nc-		0	6	19
hg-; nc+		0	0	0
hg+; nc-		0	0	0
hg+; nc+		0	0	0
				
MZ*oep*^*m*134^	GFP			
hg-; nc-		0	5	30
hg-; nc+		0	0	0
hg+; nc-		0	0	0
hg+; nc+		0	0	0
				
MZ*oep*^*m*134^	Tarama*			
hg-; nc-		0	8	15
hg-; nc+		0	1	2
hg+; nc-		5	7	7
hg+; nc+		19	5	2
				
WT	Various			
hg-; nc-		0	0	0
hg-; nc+		0	0	0
hg+; nc-		0	0	0
hg+; nc+		33	0	0

Embryos were fixed at approximately 1 dpf and processed for whole mount in situ hybridization with an antisense probes to *otx5*, *ctsl1b*, and *col2a1a*. *WT embryos are a mixture of uninjected embryos, embryos injected with 2.4 pg of mGFP mRNA and embryos injected with 2.4 pg Taram-A* mRNA. MZ*oep *embryos were injected with between 0.15 and 2.4 pg of Taram-A* mRNA, with the results for all doses grouped together or with 2.4 pg of mGFP mRNA. Whether hatching gland (hg) and notochord (nc) cells are present (+) or absent (-) is indicated.

Non-injected MZ*oep *embryos and those injected with mGFP mRNA alone expressed neither mesodermal marker, consistent with their severe lack of mesendodermal tissue (Figure 8E–E", Table 4). In contrast, 68% of embryos injected with Taram-A* mRNA had significant expression of *ctsl1b*, *col2a1a*, or both (Figure 8F–F", H-H", Table 4). Taram-A* is also capable of inducing a second axis, and as a consequence, the organization of the notochord cells was often abnormal (Figure 8H).

Importantly, closure of the anterior neural tube was corrected in 34% of the Taram-A* overexpressing embryos, as evidenced by a round-shaped pineal organ (Figure 8F–F", Table 4).

All of the embryos with a closed neural tube expressed *ctsl1b*, demonstrating that hatching gland cells were present (Figure 8F–F", Table 4). The hatching glands are derived from the anterior-most mesendodermal precursors, suggesting that anterior mesoderm is important for anterior neurulation. Most rescued embryos also expressed *col2a1a *in a group of cells that had the characteristic "stack of pennies" morphology of the notochord (Figure 8F–F", Table 4). Thus, more posterior mesoderm may also aid in closure of the anterior neural tube.

Although all embryos with a closed neural tube had rescued mesendoderm, the converse was not true. Many embryos with an elongated or divided pineal had a substantial number of notochord and hatching gland cells (Figure 8H–H", Table 4). This observation suggests that the timing or spatial aspect of mesendoderm formation may be important, which should inform future studies of neural tube closure.

## Discussion

In this study, we establish a new role for Nodal signaling in closure of the anterior neural tube in zebrafish. Further, we begin to identify important factors that may contribute to the neurulation defects in fish with deficiencies in the Nodal pathway. First, the cell adhesion molecule N-cad, which is required for zebrafish neurulation, is severely reduced at the cell membrane in MZ*oep *embryos during the stages that the neural plate is folding into a neural rod. Further, we find that cell autonomously rescuing mesoderm and endoderm formation in MZ*oep *mutants also rescues the neurulation defect. This suggests a model in which activation of the Nodal signaling pathway is required within the cells of the mesendoderm. Interactions between the mesendoderm and the overlying neural epithelium then promote neural tube closure, in part through regulating N-cad levels at the cell membrane. The NTD in zebrafish *sqt *and MZ*oep *mutants is similar to the human birth defect exencephaly, in which the rostral neural tube fails to close, ultimately resulting in anencephaly, degeneration of the developing brain, and death. N-cad and Nodals are thought to play similar roles in patterning all vertebrates, including humans [52-54]. Thus, these studies may give important insights into the causes of human NTD.

### Nodal signaling regulates closure of the anterior neural tube

The process of neurulation in zebrafish starts with the formation of the neural plate during gastrulation. The neural plate then "infolds" to form a neural keel and then a neural rod with no lumen [46,47]. The neural rod subsequently cavitates to form the neural tube [46]. During this process, the original two cell layers of the neural plate intercalate to form the single cell layer of the neural tube [28].

Recent work demonstrates that Nodal signaling is not required for neurulation of the trunk spinal cord, as this region of the neural tube is closed in MZ*oep *mutants and in WT embryos overexpressing the Nodal antagonist Lefty [55]. In contrast, we find that Nodal signaling is essential for normal closure of the rostral neural tube. As assayed by pineal anlage morphology, which we have found to be a sensitive indicator of anterior neural tube closure, all MZ*oep *mutants have an open rostral neural tube. Together, these studies demonstrate that Nodal signaling is required for an anterior-specific mechanism of neurulation.

Our data suggests that Nodal signaling is likely required during very early embryogenesis. This is illustrated by a comparison between Z*oep *and MZ*oep *mutants. Although the former has a closed neural tube, and the latter an open neural tube, they differ only in the presence and absence, respectively, of maternally supplied *oep *mRNA. Thus, maternal *oep *mRNA in Z*oep *mutants is sufficient for normal neural tube closure, suggesting that Nodal signaling before or shortly after the Midblastula transition is important for neurulation in the forebrain. The phenotype of MZ*oep *mutants is consistent with such an early role. In these mutants, the two domains of pineal precursors were typically located very laterally, suggesting that little infolding of the anterior neural plate had occurred.

### Mesoderm and anterior neurulation

Nodal signaling has multiple and complex roles during vertebrate development. We have just begun to understand how lack of Nodal signaling could result in an open neural tube. In mice, proliferation of the anterior mesenchyme helps drive shape changes in the cranial neural plate that are required for closure [30]. Our data suggests that mesoderm also has a critical role in neurulation in the rostral zebrafish neural tube. MZ*oep *mutants have only a very small amount of mesoderm in the most caudal part of their tail [34]. Rescue of mesendoderm formation in MZ*oep *mutants via overexpression of Taram-A* in turn rescued the NTD in a large number of embryos. Expression of Taram-A* cell autonomously drives cells to become mesendoderm [12,48-51]. Therefore, it is probable that Nodal signaling induces mesendoderm, and mesendoderm consequently promotes anterior neurulation. One possibility is that the mesendoderm influences the neural tube closure directly through the secretion of signaling molecules or through inducing shape changes in the neural plate. However, the mesoderm could also be acting in part or completely through other intermediate tissues. For instance, at the doses of Taram-A* used in our study, the ventral brain should also have been significantly rescued [12].

### Role of N-cad in closure of the forebrain neural tube

Our data suggests that lack of Nodal signaling ultimately decreases the expression of N-cad or its localization to the plasma membrane, as N-cad protein at the membrane is severely reduced in MZ*oep *mutants. The ordered arrangement of cells within the neural tube and the ability of the neural tube cells to form an elongated shape are also perturbed. These phenotypes are consistent with a cell adhesion defect [28,29]. Further support for a connection between Nodal signaling and N-cad comes from our rescue experiments. In a small number of cases, we were able to induce closure of the MZ*oep *neural tube solely through N-cad overexpression.

As yet, we do not understand why injection of N-cad mRNA failed to rescue neurulation in the majority of MZ*oep *fish. One possibility is that the Xenopus protein is not fully functional in zebrafish. Alternatively, N-cad mis-regulation may not the only cause of neurulation failure in MZ*oep *fish. The neural tube defects in MZ*oep *mutants are somewhat different and more severe than those found in fish that lack N-cad. For instance, the hindbrain neural tube in *n-cad *mutants has a very characteristic "T" shape that reflects the fact that the basal (ventral) neural tube forms fairly normally, while the alar (lateral/dorsal) neural tube fails to fold [28,29]. In contrast, the hindbrain neural tube of the MZ*oep *mutants was severely disrupted along the whole dorsal-ventral axis. In the most severely affected MZ*oep *mutants, the hindbrain neural tube appeared to have split along the midline. This suggests that Nodal signaling may regulate proteins in addition to N-cad, possibly even other proteins in the cadherin family. As an additional complication, there may be a very narrow range of N-cad protein that promotes neural tube closure. Too little, and the neural tube remains open, as in the zebrafish *n-cad *mutants and morphants. Overexpression or expression in the wrong place causes morphological defects (this study and [56]).

In addition, it may be difficult to rescue the MZ*oep *NTDs using mRNA. We found that N-cad mRNA levels are comparable between MZ*oep *mutants WT fish (data not shown). This suggests that the defect is primarily in the post-transcriptional regulation of N-cad. Cadherin-mediated adhesion is subject to many types of post-transcriptional regulation, including modulation by the Wnt signaling pathway and entry into recycling and degradation pathways [57,58]. Future studies will be needed to define how lack of Nodal signaling effects all of the biochemical mechanisms that control N-cad levels at the cell membrane.

### Role of N-cad in closure of the forebrain neural tube

N-cad is expressed strongly in the developing neural tube of many vertebrates, including zebrafish [29,45,56,59,60]. In *Xenopus*, downregulation of E-cadherin and upregulation of N-cad in the neural epithelium is critical for its separation from the non-neural epithelium [61]. While N-cad knockout mice have a closed neural tube, the neural tube expresses other cadherins, leading to the suggestion that a related protein(s) is sufficient to mediate the cell adhesion required for folding [62]. In zebrafish, loss of N-cad causes several NTDs that include altered cell adhesion and an open neural tube in the mid-diencephalon, midbrain, and hindbrain [29,63]. Recently, Hong and Brewster (2006) have demonstrated that N-cad is important for both the infolding of the cells at the lateral edges of the neural plate and for the radial intercalation of these cells to form a monolayer.

Here, we add to the role of N-cad in neurulation by demonstrating that it is also important for neural tube closure in the anterior diencephalon, as convergence of the pineal precursors is often incomplete in *pac *and *glo *mutants and *n-cad *morphants. The variability in the pineal phenotype was unexpected, because other defects in *pac *and *glo *mutants are completely penetrant [29,63]. One possibility is that the alleles we are using are not null mutations, and residual N-cad activity is sufficient to mediate closure in some embryos. The *pac*^*p*79*emcf *^allele causes an Isoleucine-to-Serine substitution in a conserved amino acid in the fifth cadherin extracellular (EC5) domain [64]. The EC5 domain is not essential for C-cadherin's function in cell-cell adhesion [65]. Thus, there may be some residual N-cad activity in *pac*^79*emcf*^mutants. In contrast, the *glo*^*m*117 ^allele causes a Trytophan-to-Glycine substitution in the second amino acid in the EC1 domain [66]. This Tryptophan residue is essential for cell-cell adhesion, strongly suggesting that *glo*^*m*117 ^is a null allele [66-68]. Thus, the different degrees of N-cad depletion could account for why the percentage of embryos with an elongated/divided pineal is higher in *glo*^*m*117 ^than *pac*^79*emcf *^mutants, but is unlikely to explain why the pineal phenotype is incompletely penetrant even in *glo*^*m*117 ^embryos. Closure of the anterior region of the mouse neural tube is sensitive to genetic background [30]. Thus, another possibility is that the anterior border of the NTD varies in N-cad depleted embryos due to the fact that they are not isogenic.

### Loss of ventral neural tube does not correlate with anterior NTDs

In mice and chick, the ventral neural tube forms the medial hinge point, a key site of bending in the neural plate, and also produces a signal (Sonic Hedgehog, Shh) that regulates bending at more lateral hinge points [14,16,30,69-72]. However, although Z*oep *and *cyc *mutants lack ventral brain and floor plate, we find that their anterior neural tube is closed. Thus, ventral brain is not essential for rostral neural tube closure in zebrafish. One possibility is that ventral neural tube does not play a role in closure of the zebrafish anterior neural tube. However, another possibility is that the rostral neural tube closes using an alternative pathway when the ventral neural tube is absent. The spinal cord neural tube is closed in mice mutants that lack floor plate, but the mechanism of closure is different [73-78]. In normal mice, the upper spinal cord neural tube folds by bending at the median hinge point. Ybot-Gonzalez et al. have demonstrated that in the absence of Shh signaling and formation of the medial hinge point, bending at two dorsal lateral hinge points occurs instead and is sufficient to close the neural tube [70].

### Nodal signaling in neural tube in other vertebrates

Although the Nodal signaling pathway has several well-defined and conserved functions in neural development, the role of Nodal signaling in neural tube closure remains largely unexplored. We have been unable to find any known links between Nodal and NTD in humans. However, there are some indications that Nodal signaling has a conserved role in vertebrate neurulation. Zic transcription factors act as negative regulators of *nodal *gene expression in mice and *Xenopus *[9,79]. Specifically, in mice, Zic3 acts upstream of Nodal signals in regulating left-right asymmetry of visceral organs. Interestingly, many aspects of the *zic*2 and *zic3 *mutant phenotypes are similar to those found in zebrafish Nodal signaling mutants. Holoprosencephaly, or a failure in the hemispheres of the forebrain to separate [16], is the cause of the cyclopia in *cyc*, *sqt*, and *oep *mutants. Mutations in *zic2 *in mice and humans also cause holoprosencephaly [80,81]. Most importantly for this study, both *zic3 *and *zic2 *mutant mice also have neurulation defects. Lack of Zic2 causes a delay in neurulation, spina bifida, and at a lower rate, exencephaly [80]. *zic3 *mutants have a variable defect that can include an open anterior neural tube (exencephaly and anencephaly) as well as neurulation defects in more posterior regions of the embryo [79]. It is tempting to speculate that the role of Zic2 and Zic3 in neurulation is a reflection of their role in regulation of Nodal signaling.

## Conclusion

Failures in neural tube closure are some of the most common birth defects in humans [30]. Here we have demonstrated that Nodal signaling has an essential role in the closure of the anterior neural tube. This study and those of others demonstrate that zebrafish has great potential to reveal new genes involved in neurulation, and thus new potential causes of human NTDs.

## Methods

### Zebrafish stocks

Zebrafish stocks were maintained at 28.5°C and a 14:10 hour light:dark cycle using standard methods [82]. WT (Oregon AB) and mutant embryos were produced from natural matings. Mutant alleles used included *sqt*^*cz*35 ^[40], *cyc*^*m*294 ^[39], *oep*^*m*134 ^[39], *pac*^*p*79*emcf *^[64], *glo*^*m*117 ^[66], and *flh*:eGFP1 (*flh*:eGFP1)[21]. Adult *oep*^*m*134^/*oep*^*m*134 ^fish were generated by rescuing homozygous mutants to viability by injection of *oep *mRNA at the 1–2 cell stage [19,34], and used to produce MZ*oep *embryos.

### Whole mount in situ hybridization

Whole mount RNA *in situ *hybridization was carried out using established methods [19,83]. Antisense probes included *otx5 *[20], *wnt1 *[42,43], *cone-rod homeobox *(*crx*) [84], *transducin α-subunits *(rod, *gnat1*; cone, *gnat2*) [85], *rod opsin *(*rho*) [86], *flh *[87], *epha4a*, (formerly named *receptor tyrosine kinase 1*)[88], *ctsl1b *(formerly named *hgg1*) [89], *col2a1a *[90], and *n-cad *[45].

### Immunohistochemistry

Standard fluorescent immunocytochemical methods were used for the detection of N-cad protein in 12 μm zebrafish tissue sections. The N-cad antibody was an affinity purified, rabbit polyclonal antibody prepared against zebrafish N-cad extracellular domain one [91]. Tissue sections were incubated overnight at 4°C in the N-cad antibody (6 μg/ml), followed by an anti-rabbit secondary antibody conjugated with Cy3 (1: 100 dilution; Jackson ImmunoResearch Laboratories, West Grove, PA), for 2 hours at room temperature. The sections were washed in PBS, and mounted with 90% glycerol in PBS containing 1 mM CaCl_2_.

Procedures for whole mount immunohistochemistry were described in detail previously [92]. The primary antibody zpr1 (Zebrafish International Resource Center, University of Oregon, Eugene, OR) was used at a 1:1000 dilution. A biotinylated secondary antibody (anti-mouse IgG from Vector Laboratories, Burlingame, CA) was used at 1:200. Visualization of the reaction was achieved by using a DAB kit (Vector Laboratories).

### Cryosectioning

Embryos were processed as described above for whole mount in situ hybridization and then post-fixed overnight in 4% paraformaldehyde in PBS, pH 7.4, equilibrated in 30% sucrose in DEPC PBS for twenty minutes, and transferred to 100% glucose. The embryos were decapitated to allow for easier orientation, and the heads were dipped in Tissue-Tek^® ^O.C.T. compound and positioned for transverse sections in a Pelco flat embedding chamber. The block was allowed to solidify on dry ice and then sectioned at -20°C on a Leica CM3050S cryostat.

### Morpholino injections

A translation blocking *n-cad *specific antisense morpholino oligonucleotide (*n-cad *MO 5'-TCTGTATAAAGAAACCGATAGAGTT-3'), and a standard control MO (5'-CCTCTTACCTCAGTTACAATTTATA-3', gifts from James Marrs (Indiana University) who purchased the oligonucleotides form Gene Tools, Philomath, OR, were used in the experiments. The *n-cad *MO, dissolved in Danieau buffer (58 mM NaCl, 0.7 mM KCl, 0.4 mM MgSO_4_, 0.6 mM Ca(NO_3_)_2_, 5.0 mM HEPES pH 7.6) to a concentration of 100 μM, was injected into 1–4 cell stage embryos (1–2 nl/embryo). Injection of the *n-cad *MO at this concentration was previously shown to phenocopy *pac *mutants [29], while embryos injected with the control MO were morphologically indistinguishable from uninjected WT embryos [92].

### mRNA and DNA injections

mRNA was synthesized in vivo using the mMessage Machine Kit (Ambion). To express GFP and N-cad throughout the embryo, single blastomeres of one cell stage embryos were injected with 100 pg of mGFP mRNA of the indicated amount of N-cad mRNA using a Harvard Apparatus PLI-90 nitrogen picoinjector. mGFP was expressed in a subset of cells by injecting one cell stage embryos with 100 pg of pCS2+mGFP.

To express Taram-A* in a subset of cells within the embryo, single blastomeres of 16-cell stage embryos were co-injected with a mixture of 0.15–2.4 pg of Taram-A* mRNA and 100 pg mGFP mRNA. Control embryos were injected with mGFP mRNA alone or left uninjected.

### Photography

Fluorescent and bright field images were taken on an Axiocam camera mounted on a Zeiss Axioplan 2 Imaging microscope or a SPOT camera mounted on an Olympus BX51 epifluorescence microscope. Confocal optical sections were obtained using a Leica True Confocal Scanner.

## Authors' contributions

A.A.-B. carried out the studies characterizing the pineal phenotypes and a subset of the studies characterizing the neural tube phenotype. K.I. participated in additional studies that characterized the neural tube phenotype, including the cryosectioning, the mGFP experiments, and the Taram-A* rescue experiments. Q.L. carried out the n-cad MO injections and the N-cad antibody staining. J.O.L. coordinated the study and helped carry out the mGFP and Taram-A* experiments. All authors helped draft the manuscript, and have approved the final version.

## Supplementary Material

Additional file 1 Injection of N-cad mRNA disrupts development of WT embryos. This figure shows images of mGFP (control) and N-cad mRNA injected embryos. Click here for file
