# Supplementary material for: Nodal signaling is required for closure of the anterior neural tube in zebrafish
Source: BMC Dev Biol. 2007 Nov 8;7:126. doi: 10.1186/1471-213X-7-126 (PMC2214732; doi:10.1186/1471-213X-7-126)
Supplement: Additional file 1 — Injection of N-cad mRNA disrupts development of WT embryos. This figure shows images of mGFP (control) and N-cad mRNA injected embryos. [file 1471-213X-7-126-S1.pdf]

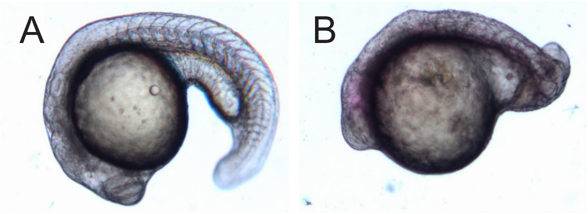

Figure Legend

Supplemental Figure 1: Injection of N-cad mRNA disrupts development of WT embryos. Embryos were injected with 75 pg of mGFP or N-cad mRNA and raised to approximately 24 hpf. Lateral views with anterior to the left. Note that the N-cad overexpressing embryo in (B) has a smaller head and shortened body axis compared to the control injected embryo in (A).
